# Mapping Fatty Acid Composition in the Human Knee: Short‐Term Repeatability at 3T

**DOI:** 10.1002/jmri.70396

**Published:** 2026-06-25

**Authors:** Dimitri Martel, Anne Adlung, Baptiste Busi, Rollanda Bernadin, Yagni Shah, Thorsten Kirsch, Richard Kijowski, Guillaume Madelin, Amparo Ruiz

**Affiliations:** ^1^ Department of Radiology NYU Grossman School of Medicine New York New York USA; ^2^ Tech4Health Institute New York New York USA; ^3^ Department of Orthopedic Surgery NYU Grossman School of Medicine New York New York USA; ^4^ Department of Biomedical Engineering NYU Tandon School of Engineering New York New York USA; ^5^ Department of Radiology Hospital for Special Surgery New York New York USA

**Keywords:** CSE‐MRI, fat composition, knee, lipids, PDFF, repeatability

## Abstract

**Background:**

Alterations in periarticular lipid composition are implicated in musculoskeletal diseases, yet short‐term reliability of MRI‐based triglyceride composition mapping in the knee is not fully established.

**Purpose:**

To evaluate 1‐week repeatability of proton‐density fat fraction (PDFF) and triglyceride fatty‐acid composition—saturated (SFA), monounsaturated (MUFA), and polyunsaturated (PUFA)—in periarticular knee tissues.

**Study Type:**

Prospective.

**Population:**

Ten healthy adults (5 female, 5 male; age 32 ± 8 years; BMI 23.5 ± 2.4 kg/m^2^).

**Field Strength/Sequence:**

3T; 12‐echo 3D spoiled gradient‐echo acquisition for chemical shift—encoded fat quantification and a proton density—weighted SPACE sequence for segmentation (0.6 mm isotropic).

**Assessment:**

Participants underwent repeated MRI 1 week apart. Femoral and tibial bone marrow, patella, Hoffa's fat pad, prefemoral fat pad, quadriceps fat pad, posterior fat pad, and subcutaneous adipose tissue were segmented and rigidly aligned. Voxelwise spectral fitting was used to estimate PDFF and fatty acid composition, including SFA, MUFA, and PUFA components. Repeatability metrics included bias, within‐subject standard deviation (wSD), within‐subject coefficient of variation (wCV%), coefficient of repeatability, and intraclass correlation coefficient (ICC).

**Statistical Tests:**

Paired *t*‐tests assessed systematic differences (*α* = 0.05); ICCs used a two‐way random‐effects, absolute‐agreement model (ICC(2,1)).

**Results:**

PDFF showed lowest variability across all regions (wCV: 1.5%–5.9%; ICC: 0.33–0.96). SFA demonstrated similar stability (wCV: 2.4%–12.6%; ICC: 0.19–0.87). MUFA exhibited anatomy‐dependent reliability (wCV: 4.1%–21.1%; ICC: 0.17–0.97), with highest repeatability in subcutaneous adipose tissue (ICC: 0.97) and Hoffa's fat pad (ICC: 0.85). PUFA displayed the greatest variability (wCV: 3.6%–52.8%; ICC: 0.10–0.94), with the greatest instability in periarticular fat pads. No paired comparisons were significant (all *p* > 0.05; range *p* = 0.14–0.98). Regional ordering remained consistent across sessions.

**Data Conclusion:**

A 12‐echo chemical shift–encoded MRI protocol provides repeatable PDFF and SFA measurements over 1 week. MUFA reliability varies by tissue, while PUFA remains least stable.

**Evidence Level:**

2 (Prospective cohort).

**Technical Efficacy Stage:**

2 (Reproducibility/feasibility evaluation).

## Plain Language Summary

Fat tissues around the knee, including bone marrow and periarticular fat pads, are active biological tissues whose composition may change in joint diseases such as osteoarthritis. We tested whether a specialized MRI technique can reliably measure fat types in these tissues. Ten healthy adults were scanned twice, 1 week apart, at 3Tesla. Total fat content and the proportions of saturated, monounsaturated, and polyunsaturated fats were measured in eight knee regions. Measurements were stable between sessions, especially for total fat content and saturated fats. This approach could support future studies tracking fat changes linked to joint disease.

## 1 Introduction

Osteoarthritis (OA) is a multifactorial joint disease characterized by progressive cartilage degeneration, subchondral bone remodeling, and inflammation of periarticular soft tissues [1, 2]. Beyond mechanical degradation, OA reflects profound metabolic and biochemical dysregulation within the joint microenvironment. The infrapatellar fat pad (IFP, Hoffa's fat pad) and subchondral bone marrow are increasingly recognized as active endocrine organs that secrete adipokines, cytokines, and lipid mediators influencing cartilage integrity and joint inflammation [3, 4, 5, 6, 7]. However, in vivo characterization of their biochemical composition remains limited, particularly regarding the distribution of specific fatty acids and their relationship to joint homeostasis.

Adipose tissue plays a central role in both systemic and local metabolism. The relative proportions of saturated (SFA), monounsaturated (MUFA), and polyunsaturated (PUFA) fatty acids (with unsaturated fatty acids defined as UFA = MUFA + PUFA) modulate membrane properties, mitochondrial function, and inflammatory signaling pathways. Altered SFA/MUFA/PUFA ratios have been linked to insulin resistance, bone fragility, and adverse tissue remodeling, including in bone marrow and periarticular depots [8, 9, 10, 11]. In musculoskeletal (MSK) disorders, compositional shifts in marrow and periarticular fat have been associated with osteoporosis and metabolic OA phenotypes, with higher saturated and lower unsaturated lipid content relating to reduced bone mineral density and impaired trabecular microarchitecture [7, 8, 9, 10]. Despite these observations, few studies have examined whether such fatty‐acid alterations can be robustly detected and quantified in the knee joint [12, 13].

Magnetic resonance imaging (MRI) provides a noninvasive platform to assess both structural and compositional properties of MSK tissues. Quantitative biomarkers such as T_2_, T_2_*, T_1_ρ, and diffusion tensor imaging (DTI) probe water‐associated contrast mechanisms related to matrix hydration, collagen organization, and microstructural anisotropy [14, 15, 16], but they offer limited direct sensitivity to lipid metabolism. Chemical shift–encoded MRI (CSE‐MRI) exploits the phase evolution of water and fat resonances across multiple echoes, enabling quantitative fat–water separation and advanced spectral modeling. Initially developed and validated for hepatic fat quantification [17, 18, 19, 20], CSE‐MRI has been extended to bone marrow and other MSK depots, including vertebral and proximal femur marrow, where it can detect changes in marrow adipose tissue (MAT) composition and link them to metabolic or pharmacologic perturbations [8, 9, 10, 21].

Recent advances in spectrum‐modeled multi‐echo CSE‐MRI now allow in vivo discrimination between triglyceride pools that differ in degree of saturation by jointly estimating PDFF and fatty‐acid composition, including SFA, MUFA, and PUFA fractions [11, 21, 22, 23, 24, 25, 26]. These approaches have been validated against MR spectroscopy and, in some settings, biochemical analysis in liver, adipose tissue, skeletal muscle, and bone marrow, and have been applied to abdominal adipose depots in humans [11, 21, 22, 24, 25], but have not yet been systematically evaluated in periarticular knee tissues.

Applying CSE‐MRI to the knee offers an opportunity to investigate regional lipid remodeling within periarticular tissues that are increasingly implicated in OA pathogenesis. The IFP demonstrates phenotypic and compositional changes under mechanical loading and systemic metabolic influence, with reported associations to synovitis, cartilage damage, and symptom burden [3, 5, 6, 7]. Similarly, bone marrow adiposity is emerging as a key regulator of subchondral bone quality and osteochondral coupling, with marrow fat composition linked to bone fragility and altered remodeling [8, 9, 10]. Quantitative mapping of PDFF and fatty‐acid composition in these compartments may therefore provide complementary biomarkers to traditional cartilage‐ and fluid‐based MRI parameters, enabling a more comprehensive characterization of the joint environment in both health and disease.

Despite these advances, the repeatability and regional variability of fatty‐acid composition mapping in the knee have not been systematically established. Demonstrating short‐term reproducibility is important for interpreting longitudinal imaging changes, defining minimal detectable differences, and designing adequately powered clinical studies [20, 27, 28, 29, 30]. While PDFF repeatability is well characterized in hepatic and marrow applications [17, 20], repeatability metrics for SFA, MUFA, and PUFA in small periarticular structures remain unknown. Because metabolic remodeling in periarticular tissues is thought to contribute to symptom progression, inflammation, and treatment responsiveness, the reliability of compositional fat imaging determines whether changes over time reflect biology or measurement noise. Establishing test–retest benchmarks is therefore a prerequisite for clinical translation. Therefore, the purpose of this study was threefold:

(i) to implement a high‐resolution, 12‐echo CSE‐MRI protocol at 3T for quantitative mapping of PDFF and fatty‐acid composition (SFA, MUFA, PUFA) in the human knee; (ii) to evaluate short‐term test–retest repeatability across femoral and tibial bone marrow, patella, Hoffa's fat pad, prefemoral fat pad, quadriceps fat pad, posterior fat pad, and subcutaneous adipose tissue; and (iii) to characterize regional lipid composition and repeatability metrics to support the use of compositional fat imaging in longitudinal and interventional MSK research.

## 2 Materials and Methods

### 2.1 Study Design and Participants

Ten healthy adult volunteers were scanned twice, 1 week apart (TP_1_ and TP_2_), with all acquisitions performed on the right knee. The study was approved by the institutional review board, and written informed consent was obtained from all participants. Participants were recruited as healthy volunteers from the local community; none were imaged for clinical indications. Inclusion criteria were: age 18–55 years and body mass index 18.5–30.0 kg/m^2^. Exclusion criteria were: prior knee trauma, surgery, or diagnosis of MSK disease; contraindications to MRI; pregnancy; and any systemic metabolic disorder known to alter bone marrow or adipose tissue composition. No formal sample size calculation was performed; 10 participants is consistent with pilot repeatability studies in quantitative MSK MRI and was primarily constrained by scan availability.

### 2.2 MRI Acquisition

All MRI examinations were performed on a 3T Prisma system (Siemens Healthineers, Erlangen, Germany) using a 15‐channel knee coil. A 3D proton density–weighted fast spin‐echo (SPACE) sequence was acquired for high‐resolution anatomical coverage of the entire joint (voxel size = 0.6 mm isotropic; TR/TE = 900/28 ms). These images were used for segmentation and spatial reference. Quantitative CSE‐MRI was performed using a multi‐echo 3D spoiled gradient‐echo acquisition for fat–water separation and fatty‐acid quantification. Imaging parameters were: TE_1_ = 1.2 ms, ΔTE = 1.2 ms, 12 echoes, TR/FA = 16 ms/5°, bandwidth = 2000 Hz/pixel, 64 sagittal slices, and voxel size = 0.5 × 0.5 × 3.0 mm^3^. The slab was centered between the femoral condyles to include the femorotibial and patellofemoral compartments and Hoffa's fat pad.

### 2.3 Image Reconstruction and Preprocessing

Magnitude and phase images for all echoes were exported in DICOM format and combined to generate complex data for each echo. Reconstruction was performed using custom Python routines (NumPy/SciPy), which applied standard corrections for background phase and spatial field inhomogeneity. The phase of each echo was unwrapped using a Laplacian‐based algorithm, and residual B_0_‐related phase evolution was removed through voxelwise demodulation. These steps yielded a corrected complex multi‐echo signal suitable for quantitative fat–water modeling and fatty‐acid spectral fitting, consistent with prior composition‐mapping workflows [12, 21, 24].

### 2.4 Fatty‐Acid Composition Modeling

The voxel‐wise multi‐echo signal was modeled as the superposition of water and fat components modulated by a joint effective transverse relaxation rate and local off‐resonance:

$$S\left(\mathrm{TE}\right)=\left[\rho_{W}+\rho_{F}\sum_{p=1}^{N_{p}}\alpha_{p}e^{i2\pi \Delta f_{p}\mathrm{TE}}\right]\;e^{-R_{2}^{*\mathrm{TE}}}\;e^{i2\pi \Delta B_{0}\mathrm{TE}}$$

where $\rho_{W}$ and $\rho_{F}$ are the complex amplitudes of water and fat, $R_{2}^{*}$ is a joint water–fat effective transverse relaxation rate (s^−1^), $\alpha_{p}$ is the relative amplitude of the *p*th fat resonance, $\Delta f_{p}$ is its frequency offset from water (Hz), and $\Delta B_{0}$ is the local field inhomogeneity (Hz).

### 2.5 Triglyceride Spectral Model

The fat signal was modeled using a multi‐peak triglyceride spectrum comprising nine ^1^H resonances referenced to the water peak at 4.7 ppm, following established lipid spectroscopy and chemical‐shift–encoded MRI frameworks [24, 26, 31]. Each resonance corresponds to a specific proton environment within the triglyceride molecule—such as terminal methyl, backbone methylene, allylic, bis‐allylic, carbonyl‐adjacent, and glycerol‐bound protons—with peak amplitudes determined directly by the number of contributing protons (Table 1).

**TABLE 1:** Fat resonances included in the triglyceride spectral model used for multi‐echo chemical shift–encoded MRI fat quantification. Chemical shift values, structural assignments, theoretical amplitudes, and empirical relative amplitudes are shown for the 9‐peak triglyceride spectral model used for PDFF and fatty‐acid composition estimation.

Peak/group	Chemical shift (ppm)	Structural environment	Theoretical amplitude	Relative amplitude
CH_3_ terminal	0.9	Terminal methyl	9	0.09
(CH_2_)n	1.3	Methylene backbone	6× (CL−4)−8 × ndb + 2 × nmidb	0.70
β‐CH_2_	1.6	β‐methylene adjacent to acyl chain	6	0.10
Allylic CH_2_	2.1	Adjacent to double bond	4× (ndb−nmidb)	0.06
Acyl CH_2_	2.3	Adjacent to carbonyl/acyl linkage	6	0.005
Bis‐allylic CH_2_	2.8	Between double bonds	2 × nmidb	0.02
Glycerol CH_2_	4.2	Triglyceride backbone	4	0.005
Glycerol CH	5.2	Triglyceride backbone	1	0.005
Olefinic CH=CH	5.3	Unsaturated carbons	2 × ndb	0.02

Abbreviations: CH_2_, methylene group; CH_3_, methyl group; CL, chain length; CSE‐MRI, chemical shift–encoded magnetic resonance imaging; ndb, number of double bonds; nmidb, number of methylene‐interrupted double bonds.

The *theoretical amplitude* $A_{p}$ for each peak is expressed analytically in terms of fatty‐acid chain length (CL), number of double bonds (ndb), and number of methylene‐interrupted double bonds (nmidb). This formulation captures olefinic, bis‐allylic, allylic, carbonyl‐adjacent, terminal methyl, backbone methylene, and glycerol‐backbone protons and allows the model to explicitly encode unsaturation chemistry. Normalized weights for the signal model are: $\alpha_{p}=\frac{A_{p}}{\sum_{k}A_{k}}$.

### 2.6 Model Fitting and Quantification

Nonlinear least‐squares optimization was applied voxel‐wise to estimate $\rho_{W}$,$\rho_{F}$, $R_{2}^{*}$, and $\Delta B_{0}$. Initial parameter guesses were derived from a complex Dixon decomposition to improve convergence.

From the fitted spectrum, three structural characteristics of the triglyceride chains were derived [13, 24]: mean number of double bonds per chain (ndb), mean number of methylene‐interrupted double bonds (nmidb), and mean chain length (CL, number of CH_2_ units per fatty acid). These parameters were constrained within physiologic ranges derived from prior marrow and adipose‐tissue studies to stabilize the fit [12, 13]. Voxel‐wise fatty‐acid fractions were computed as follows:

$$F_{\text{PUFA}}=\frac{\text{nmidb}}{3},\;F_{\text{MUFA}}=\frac{\mathrm{ndb}-2\times \text{nmidb}}{3},\;F_{\mathrm{SFA}}=1-\left(F_{\text{PUFA}}+F_{\text{MUFA}}\right),$$

and normalized such that

$$F_{\mathrm{SFA}}+F_{\text{MUFA}}+F_{\text{PUFA}}=1$$

PDFF was defined as follows:

$$\text{PDFF}=\frac{\left|\rho_{F}\right|}{\left|\rho_{W}\right|+\left|\rho_{F}\right|}\times 100\%.$$

This model establishes a direct link between MR phase evolution and the molecular composition of triglycerides, enabling quantitative mapping of proton‐density fat fraction (PDFF) and fatty‐acid composition (SFA, MUFA, PUFA). It extends methods validated in hepatic [17, 26], marrow [12, 13], and muscle tissues to the periarticular knee environment.

### 2.7 ROI Segmentation and Quantification

Eight regions of interest (ROIs)—femoral bone marrow, tibial bone marrow, patella, Hoffa's fat pad, prefemoral fat pad, quadriceps fat pad, posterior fat pad, and subcutaneous adipose tissue—were semi‐automatically delineated by one researcher (D.M., 10+ years of quantitative MSK MRI experience including 2 years focused on knee imaging) on the 3D proton density–weighted SPACE using ITK‐SNAP with nnInteractive assistance [32]. The interactive learning tool accelerated boundary identification while preserving complete user control over the final masks. All ROIs were then visually inspected and manually refined to ensure anatomical accuracy and boundary consistency across timepoints.

To ensure spatial correspondence between segmentations and the quantitative CSE‐MRI maps, each ROI mask was registered to the multi‐echo dataset using rigid transformation computed with ANTs (Advanced Normalization Tools). A rigid‐only approach was selected to avoid altering the biological geometry of the ROIs; deformable models can introduce nonphysical shape changes that compromise quantitative fat measurements, particularly in small marrow regions and Hoffa's fat pad. Because all acquisitions were performed within the same session and with consistent positioning, rigid alignment provided sufficient correction of residual in‐plane shifts and rotations without modifying ROI morphology. Rigid registration was performed with ANTs [33] using the antsRegistration framework and a mutual‐information similarity metric with multiresolution optimization (Gaussian smoothing and image downsampling) to provide robust alignment across contrasts. The resulting rigid transform was then applied to resample each ROI mask into the multi‐echo CSE‐MRI space using nearest‐neighbor interpolation to preserve discrete label values.

### 2.8 Repeatability Analysis

Repeatability between TP_1_ and TP_2_ was evaluated for each ROI and parameter (PDFF, SFA, MUFA, PUFA). Metrics included: Bias (Δmean) = average difference between TP_2_ and TP_1_, within‐subject standard deviation (wSD) = SD of paired differences/√2, coefficient of repeatability (CR) = 2.77 × wSD (95% limits of agreement), within‐subject coefficient of variation (wCV %) = 100 × (wSD/overall mean), and intraclass correlation coefficient [ICC(2,1)] with 95% confidence intervals. Bland–Altman and boxplot visualizations were used to confirm consistency across timepoints.

### 2.9 Statistical Analysis

All calculations were performed in Python (NumPy, SciPy, Statsmodels, Matplotlib). ICC confidence intervals were computed using the F‐distribution for a two‐way random, absolute‐agreement model. Statistical significance was defined as *p* < 0.05. Significance of systematic inter‐session bias was assessed using paired *t*‐tests (*α* = 0.05).

## 3 Results

### 3.1 Segmentation and Image Reconstruction

**FIGURE 1:**
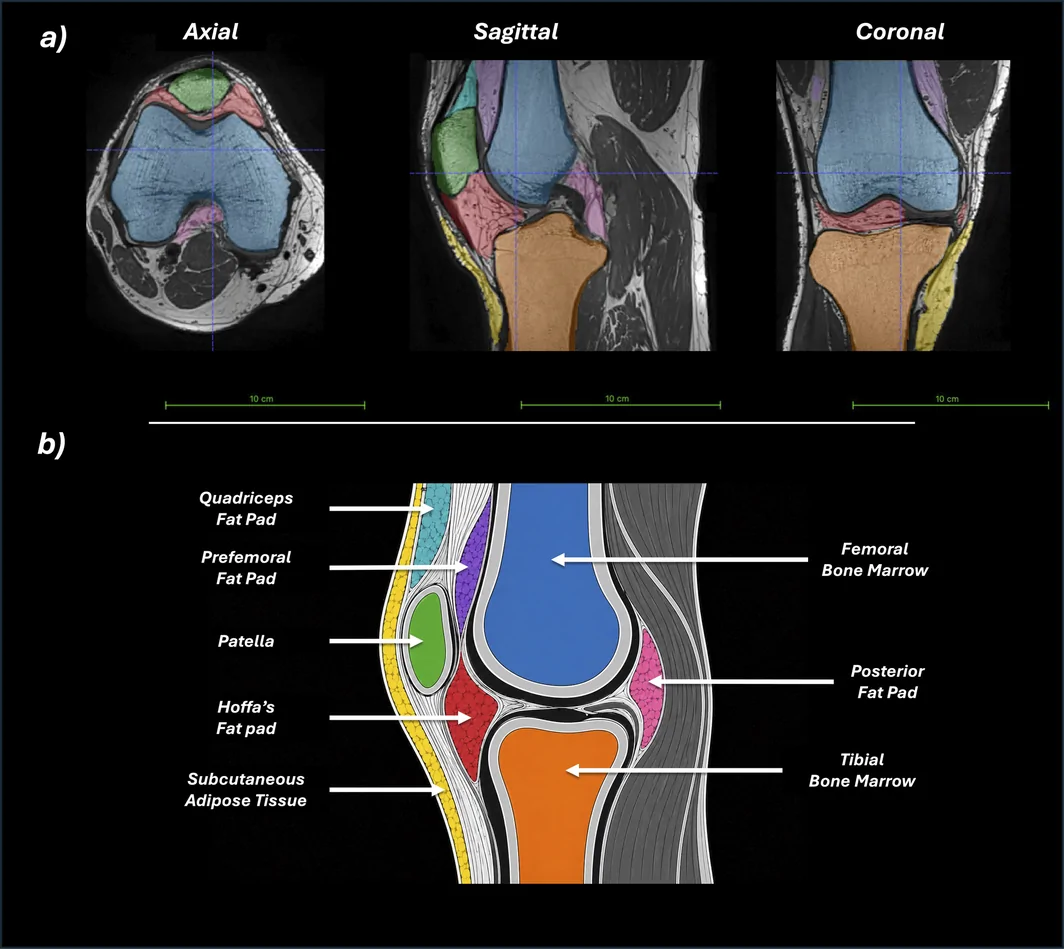
(a) Representative axial, sagittal, and coronal PD‐weighted SPACE images with color‐coded segmentation overlays illustrating the eight periarticular knee regions of interest. Crosshairs indicate the shared reference slice position across views. (b) Anatomical schematic of the knee in sagittal cross‐section showing the spatial arrangement of all eight ROIs: Femoral bone marrow (blue), prefemoral fat pad (purple), quadriceps fat pad (teal), patella (green), Hoffa's fat pad (red), posterior fat pad (pink), tibial bone marrow (orange), and subcutaneous adipose tissue (yellow). ROIs were semi‐automatically delineated on PD‐SPACE images using nnInteractive‐assisted segmentation in ITK‐SNAP by one researcher (D.M.), then visually reviewed and manually refined, and rigidly registered to the CSE‐MRI space to ensure consistent boundaries for test–retest analyses.

**FIGURE 2:**
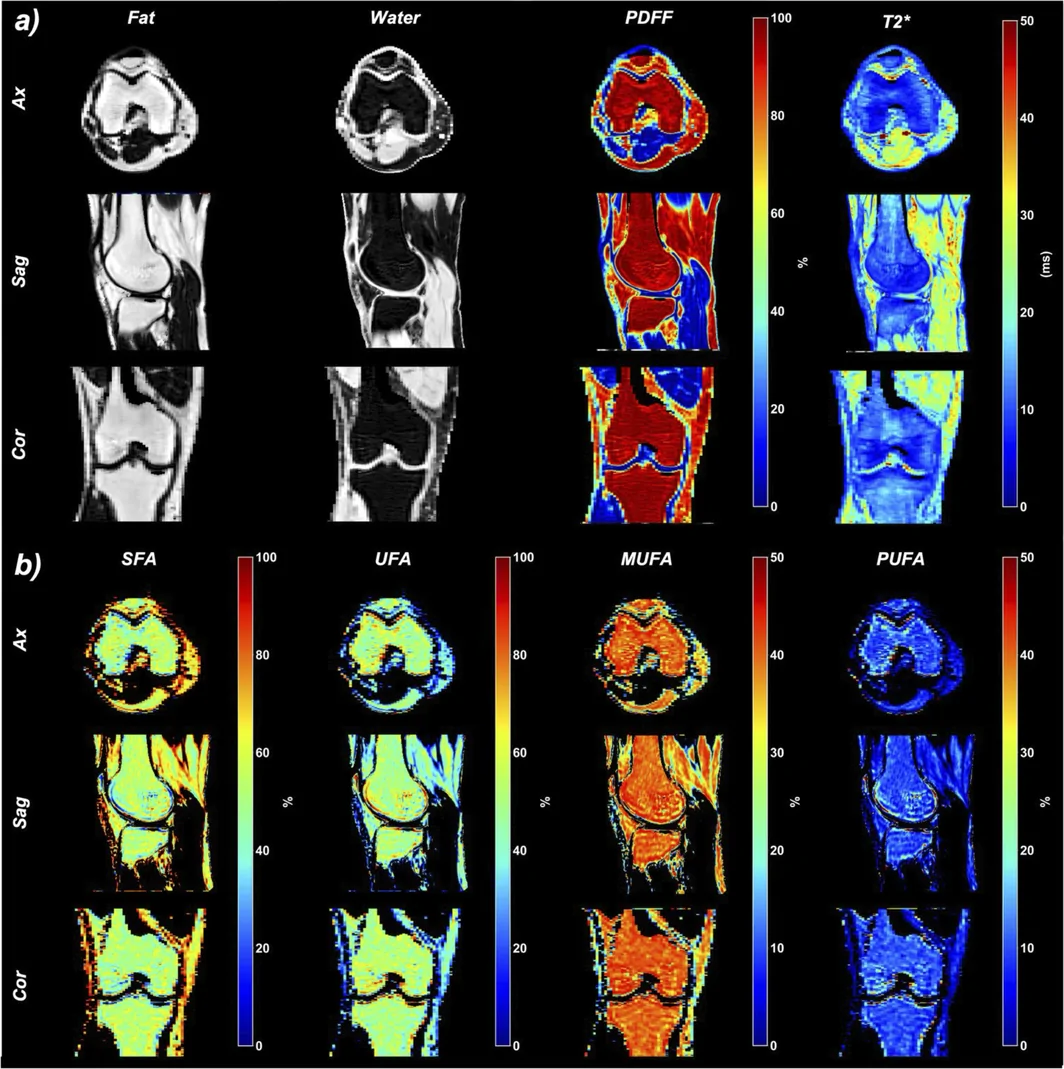
(a) Reconstructed fat, water, PDFF, and T_2_* maps demonstrate clear contrast between bone marrow and surrounding soft tissues across axial, sagittal, and coronal planes. (b) Voxel‐wise maps of saturated (SFA), unsaturated (UFA = MUFA + PUFA), mono‐unsaturated (MUFA), and poly‐unsaturated (PUFA) fatty acids show distinct spatial patterns and good overall signal uniformity across the knee, illustrating the feasibility of chemical‐shift‐encoded mapping of individual fatty‐acid components.

Ten healthy adult volunteers were enrolled (5 female, 5 male; age 32 ± 8 years; BMI 23.5 ± 2.4 kg/m^2^; height 174 ± 7 cm; weight 72 ± 8 kg). All completed both scan sessions without adverse events or data loss. Semi‐automatic segmentation of all eight ROIs was completed successfully in all 10 subjects at both timepoints (Figure 1). Apparent SNR at echo 1 (defined as mean ROI signal divided by the standard deviation of signal in a background corner region positioned far from the knee coil) ranged from 171 ± 74 (posterior fat pad) to 390 ± 144 (subcutaneous adipose tissue), with an overall mean of 249 ± 100 across all ROI–subject combinations. Fat‐dominant soft‐tissue ROIs (Hoffa's fat pad, prefemoral fat pad, quadriceps fat pad, posterior fat pad, subcutaneous adipose tissue) showed increasing apparent SNR toward later echoes, consistent with their long effective T2*, whereas bone marrow and patella apparent SNR decreased with echo time due to accelerated signal decay from trabecular bone. The nnInteractive‐assisted workflow produced consistent 3D masks across timepoints without failed or incomplete segmentations. CSE‐MRI reconstruction generated spatially coherent fat, water, PDFF, T_2_*, and fatty‐acid fraction (SFA, MUFA, PUFA) maps throughout the knee (Figure 2), with visually uniform signal profiles and no qualitative artifacts affecting the targeted ROIs.

### 3.2 Test–Retest Repeatability

Repeatability metrics across all eight ROIs are summarized in Table 2. PDFF showed the lowest overall variability, with wCV ranging from 1.49% (patella) to 5.91% (subcutaneous adipose tissue) and ICC from 0.33 (prefemoral fat pad) to 0.96 (patella). Femoral and tibial bone marrow showed the lowest absolute wCV values (1.57% and 1.98%, respectively) with moderate ICCs (0.47 and 0.50); this pattern reflects restricted between‐subject variance in this healthy cohort rather than poor absolute repeatability, and the corresponding CR values (3.81% and 4.79%) are consistent with reliable measurement. SFA demonstrated comparable repeatability across ROIs (wCV: 2.40%–12.56%; ICC: 0.19–0.87), with highest ICCs in Hoffa's fat pad (0.87) and prefemoral fat pad (0.87), and greatest instability in quadriceps fat pad (wCV = 12.56%; ICC = 0.33). MUFA showed region‐dependent repeatability (wCV: 4.11%–21.13%; ICC: 0.17–0.97), with highest ICCs in subcutaneous adipose tissue (0.97) and Hoffa's fat pad (0.85), and greatest instability in quadriceps fat pad (wCV = 21.13%). PUFA exhibited the widest variability (wCV: 3.57%–52.79%; ICC: 0.10–0.94), with lowest repeatability in posterior fat pad (wCV = 52.79%) and quadriceps fat pad (wCV = 35.63%), consistent with the lower spectral amplitude of PUFA resonances and greater sensitivity to field inhomogeneity in these regions.

**TABLE 2:** Test–retest repeatability metrics for proton‐density fat fraction and triglyceride fatty‐acid composition across periarticular knee regions. Mean values, bias, within‐subject standard deviation, coefficient of repeatability, within‐subject coefficient of variation, and intraclass correlation coefficient are reported for femoral bone marrow, tibial bone marrow, patella, Hoffa's fat pad, prefemoral fat pad, quadriceps fat pad, posterior fat pad, and subcutaneous adipose tissue.

ROI	Map	Mean (%)	Bias (%)	wSD	CR	wCV (%)	ICC (95% CI)
Femoral bone marrow	PDFF	87.79	−0.05	1.37	3.79	1.57	0.47 (−0.25, 0.84)
	SFA	47.10	−0.51	1.25	3.46	2.65	0.67 (0.13, 0.91)
	MUFA	36.00	−0.56	1.61	4.46	4.46	0.17 (−0.51, 0.70)
	PUFA	11.35	+0.29	0.41	1.14	3.57	0.85 (0.52, 0.96)
Tibial bone marrow	PDFF	87.14	−0.18	1.73	4.79	1.98	0.50 (−0.20, 0.85)
	SFA	49.30	+0.34	1.65	4.57	3.35	0.66 (0.08, 0.90)
	MUFA	34.53	−0.47	1.42	3.93	4.11	0.40 (−0.27, 0.81)
	PUFA	9.89	−0.14	0.73	2.02	7.40	0.55 (−0.11, 0.87)
Patella	PDFF	74.78	+0.40	1.11	3.07	1.49	0.96 (0.84, 0.99)
	SFA	38.15	+0.29	2.25	6.23	5.89	0.84 (0.42, 0.96)
	MUFA	29.93	−0.03	2.13	5.90	7.11	0.66 (0.00, 0.91)
	PUFA	13.95	−0.20	1.50	4.16	10.76	0.57 (−0.15, 0.89)
Hoffa's fat pad	PDFF	79.13	+1.33	2.54	7.04	3.21	0.65 (0.13, 0.90)
	SFA	49.06	−0.20	1.30	3.60	2.65	0.87 (0.57, 0.97)
	MUFA	31.12	+0.13	1.35	3.74	4.33	0.85 (0.49, 0.96)
	PUFA	8.89	+0.35	0.51	1.41	5.75	0.94 (0.78, 0.98)
Prefemoral fat pad	PDFF	81.57	+1.70	3.21	8.89	3.93	0.33 (−0.29, 0.77)
	SFA	54.69	+0.02	1.31	3.63	2.40	0.87 (0.55, 0.97)
	MUFA	23.92	+1.30	2.89	8.01	12.10	0.46 (−0.18, 0.83)
	PUFA	4.16	+0.50	1.08	2.99	26.10	0.10 (−0.52, 0.66)
Quadriceps fat pad	PDFF	69.31	−3.58	3.43	9.50	4.95	0.78 (0.26, 0.94)
	SFA	56.62	−0.37	7.11	19.69	12.56	0.33 (−0.48, 0.80)
	MUFA	25.93	−2.45	5.48	15.18	21.13	0.52 (−0.14, 0.87)
	PUFA	5.40	−0.18	1.93	5.35	35.63	0.74 (0.18, 0.94)
Posterior fat pad	PDFF	70.91	+0.03	2.17	6.01	3.06	0.87 (0.54, 0.96)
	SFA	49.23	−1.45	4.40	12.19	8.94	0.19 (−0.50, 0.72)
	MUFA	20.93	+1.00	1.94	5.37	9.29	0.85 (0.54, 0.96)
	PUFA	4.40	+1.34	2.32	6.43	52.79	0.14 (−0.44, 0.67)
Subcutaneous adipose tissue	PDFF	90.03	−1.07	5.32	14.74	5.91	0.65 (0.06, 0.90)
	SFA	52.85	−0.45	2.95	8.17	5.58	0.81 (0.40, 0.95)
	MUFA	34.51	+0.16	1.63	4.52	4.72	0.97 (0.89, 0.99)
	PUFA	7.87	+0.68	1.32	3.66	16.79	0.84 (0.49, 0.96)

Abbreviations: CI, confidence interval; CR, coefficient of repeatability (2.77 × wSD); ICC, intraclass correlation coefficient; MUFA, monounsaturated fatty acid fraction; PDFF, proton‐density fat fraction; PUFA, polyunsaturated fatty acid fraction; ROI, region of interest; SFA, saturated fatty acid fraction; wCV, within‐subject coefficient of variation; wSD, within‐subject standard deviation.

Across all 32 parameter–ROI combinations, paired comparisons between TP_1_ and TP_2_ showed no significant differences (all *p* > 0.05; range *p* = 0.14–0.98), consistent with low absolute inter‐session biases across all ROIs (−1.45% to +1.70%).

### 3.3 Agreement and Distribution

**FIGURE 3:**
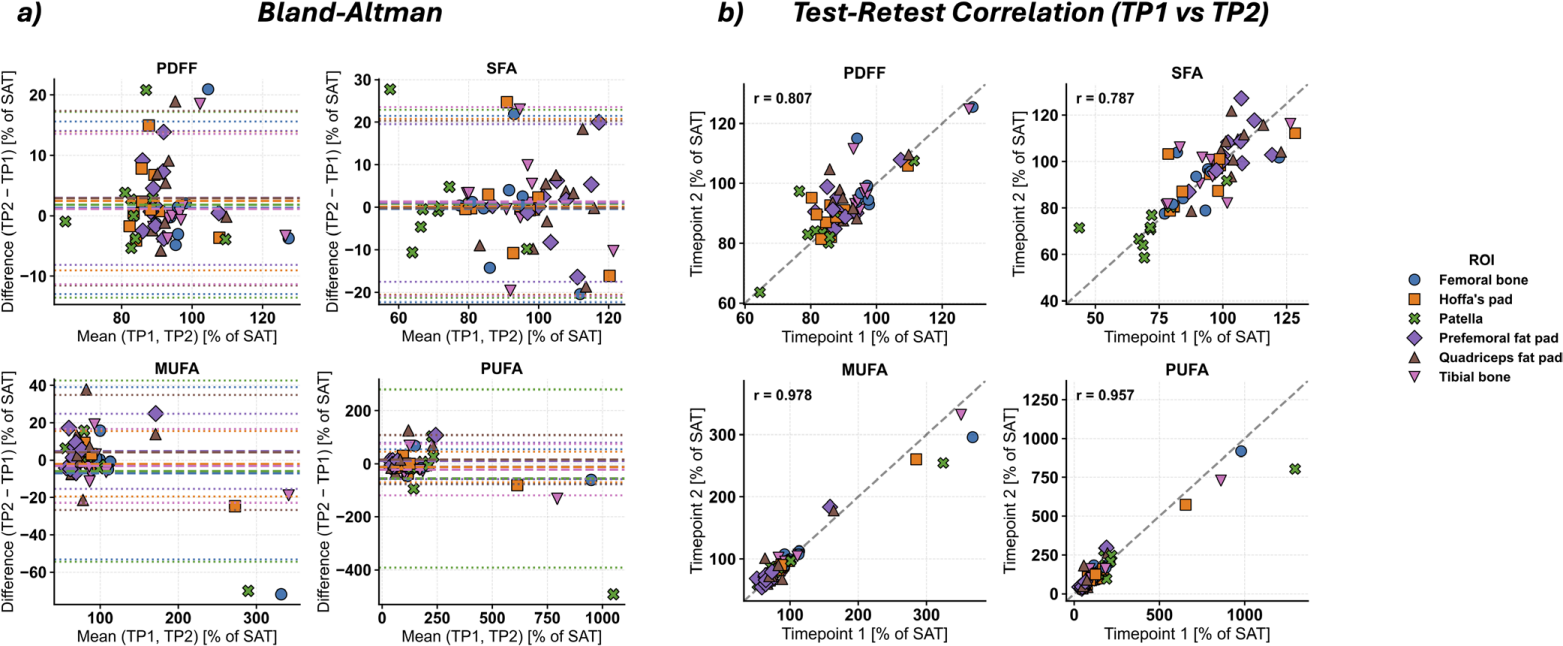
(a) Bland–Altman plots and (b) test–retest correlations for PDFF, SFA, MUFA, and PUFA across eight regions. Mean bias was below 2% for most ROI–parameter combinations. Pearson correlation coefficients were *r* = 0.90 (PDFF), *r* = 0.80 (SFA), *r* = 0.87 (MUFA), and *r* = 0.88 (PUFA), confirming low inter‐session drift across all ROIs.

**FIGURE 4:**
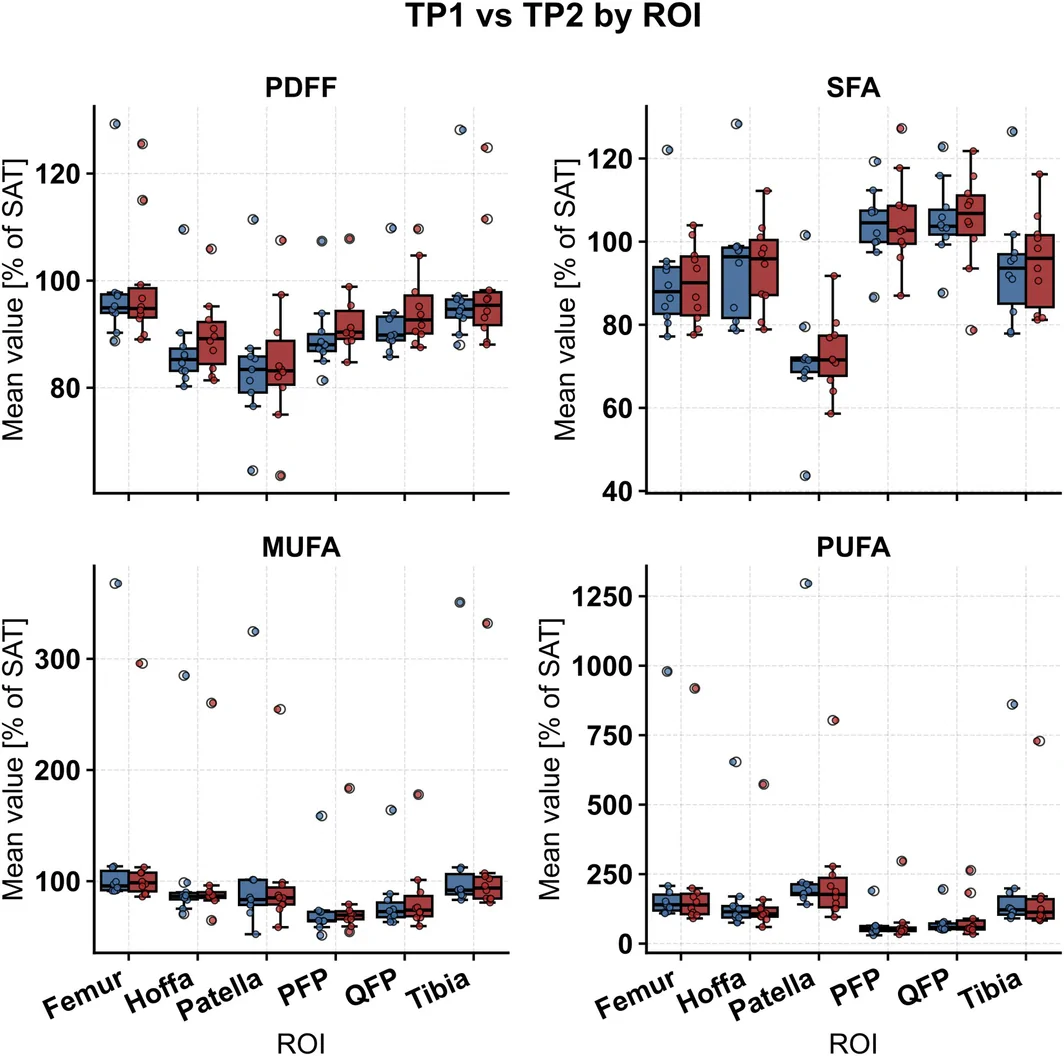
Boxplots comparing TP_1_ and TP_2_ mean values across all eight ROIs show overlapping distributions and stable medians between sessions. PDFF is highest in subcutaneous adipose tissue and bone marrow compartments; SFA predominates across all regions while MUFA and PUFA fractions are lower in periarticular fat pads than in subcutaneous adipose tissue.

Bland–Altman plots (Figure 3) demonstrated mean biases below 2% for most ROI–parameter combinations, with wider limits of agreement in PUFA‐rich periarticular fat pads and subcutaneous adipose tissue, without systematic proportional error. Test–retest scatter plots (Figure 3) showed high overall correlations across all ROIs combined (*r* = 0.88 for PDFF, *r* = 0.87 for SFA, *r* = 0.90 for MUFA, *r* = 0.88 for PUFA). TP_1_–TP_2_ boxplots (Figure 4) showed overlapping medians and interquartile ranges in all ROIs, supporting consistent distributional characteristics between visits.

### 3.4 Regional Lipid Composition

Regional mean values (Table 2) demonstrated consistent spatial ordering across timepoints. PDFF was highest in subcutaneous adipose tissue (90.0%), followed by femoral and tibial bone marrow (87.8% and 87.1%), prefemoral fat pad (81.6%), Hoffa's fat pad (79.1%), patella (74.8%), posterior fat pad (70.9%), and quadriceps fat pad (69.3%). Subcutaneous adipose tissue served as a compositional reference, exhibiting high SFA (52.9%), high MUFA (34.5%), and the highest MUFA ICC in the dataset (0.97). Across all compartments, SFA constituted the largest lipid fraction (range: 38%–57%), followed by MUFA (21%–36%) and PUFA (4%–14%), with consistent relative ordering between sessions. Periarticular fat pads showed lower MUFA and PUFA fractions than subcutaneous adipose tissue, reflecting compositional differences between depot types.

## 4 Discussion

This study examined short‐term test–retest repeatability of PDFF and fatty‐acid composition mapping in periarticular knee tissues using a 12‐echo CSE‐MRI protocol at 3T. Compositional estimates were obtained across eight regions: femoral and tibial bone marrow, patella, Hoffa's fat pad, prefemoral fat pad, quadriceps fat pad, posterior fat pad, and subcutaneous adipose tissue. PDFF and the dominant triglyceride fraction (SFA) demonstrated low within‐subject variability across all regions, whereas MUFA repeatability depended on tissue type and PUFA exhibited the largest dispersion, particularly in periarticular fat pads. These findings help to characterize repeatability benchmarks needed to interpret longitudinal change, differentiate biological variability from measurement noise, and design properly powered MSK studies.

The repeatability of PDFF in this study aligns with prior hepatic and marrow applications of CSE‐MRI, supporting its robustness even in smaller periarticular structures and in proximity to cartilage–bone interfaces [17, 20, 21]. SFA behaved similarly, likely due to its prominent spectral contribution and comparatively high signal‐to‐noise behavior. MUFA demonstrated region‐dependent performance; estimates were more stable in Hoffa's fat pad and patella, but lower in marrow compartments. This pattern is consistent with susceptibility and trabecular geometry effects known to influence signal stability and voxel composition in bone marrow [8, 10]. PUFA exhibited the widest variability, in agreement with reports that low‐abundance resonances are more sensitive to echo timing, field perturbations, and fitting instability [12, 23]. Together, these observations suggest that compositional metrics derived from unsaturated lipid pools may require tailored acquisition or reconstruction strategies to improve precision.

Regional lipid distributions followed expected physiological hierarchies. PDFF was greatest in marrow compartments and lowest in patella, reflecting known differences in adipocyte density and loading environment [7, 8]. SFA predominance across all regions agrees with marrow and adipose spectroscopy studies [21, 24]. In the present study, femoral and tibial bone marrow SFA averaged 47% and 49%, respectively, consistent with prior CSE‐MRI marrow studies reporting SFA of 45%–52% [12, 21, 24]. Hoffa's fat pad SFA (49%) and subcutaneous adipose tissue SFA (53%) fall within the range reported for periarticular and subcutaneous depots in prior spectroscopy and CSE‐MRI studies [11, 24]. MUFA and PUFA showed consistent spatial ordering across timepoints, indicating that periarticular fat composition is stable in healthy adults over short intervals.

Technical considerations likely contributed to variation observed across metrics. Susceptibility gradients and partial‐volume effects at trabecular interfaces can disproportionately influence unsaturated lipid estimates. The joint R_2_* model applied here reduces instability but does not differentiate separate relaxation behavior for fat and water, which may introduce uncertainty when fat content is low [12]. Phase‐based estimation preserves spectral fidelity but remains sensitive to field drift and to local geometry near bone surfaces [8, 12]. Additional optimization—such as improved denoising, longer echo sampling, adaptive echo placement, or regularized spectral fitting—may particularly benefit PUFA estimation. The design of this work supports practical interpretation. PDFF and SFA demonstrated variability consistent with detection of moderate biological change over short periods, supporting their potential use as quantitative imaging endpoints in metabolic, MSK, or interventional research. MUFA and PUFA may contribute mechanobiological or biochemical insight but require cautious interpretation in marrow‐rich regions, particularly where expected changes are modest. Compositional fat mapping extends established MSK MRI biomarkers by probing slower metabolic remodeling mechanisms rather than matrix‐based water contrast [15, 16], suggesting complementary value in studies of OA, obesity, and systemic metabolic disease.

## 5 Limitations

Participants were healthy young adults scanned over a short interval, so results may not generalize to older, obese, or osteoarthritic populations that exhibit different marrow composition or turnover. ROI‐based evaluation does not capture voxelwise gradients or spatial heterogeneity. PUFA estimation may benefit from optimized acquisition timing, increased signal‐to‐noise ratio, or regularized spectral fitting. Semi‐automated segmentation was used; future work may integrate fully automated deep learning parcellation to improve scalability. Longitudinal studies should evaluate whether observed repeatability supports sensitivity to clinical endpoints such as pain trajectories, marrow lesion dynamics, or metabolic change.

## 6 Conclusion

A 12‐echo CSE‐MRI protocol at 3T produced repeatable maps of PDFF and triglyceride fatty‐acid composition across eight periarticular and reference knee regions. PDFF and SFA exhibited low test–retest variability, supporting their use as quantitative endpoints in MSK research. MUFA showed region‐dependent repeatability, and PUFA demonstrated the greatest variability, particularly in periarticular fat pads, consistent with lower spectral signal contribution and fitting sensitivity. Regional lipid distributions were stable across timepoints, with distinct compositional profiles across marrow, fat pad, and subcutaneous adipose compartments. These findings establish repeatability benchmarks that support pilot applications of compositional fat MRI as a quantitative biomarker in metabolic, inflammatory, and OA‐related MSK research.

## Funding

This work was supported by NIH R01 AR082225, R01 AR079182, R01 AR082670 and was performed under the rubric of the Center for Advanced Imaging Innovation and Research (CAI2R, https://www.cai2r.net), an NIBIB National Center for Biomedical Imaging and Bioengineering (NIH P41 EB017183).

## Conflicts of Interest

The authors declare no conflicts of interest.
